# Synergistic Activity of *Gloeophyllum striatum*-Derived AgNPs with Ciprofloxacin and Gentamicin Against Human Pathogenic Bacteria

**DOI:** 10.3390/ijms26083529

**Published:** 2025-04-09

**Authors:** Aleksandra Tończyk, Katarzyna Niedziałkowska, Przemysław Bernat, Katarzyna Lisowska

**Affiliations:** 1 Department of Industrial Microbiology and Biotechnology, Faculty of Biology and Environmental Protection, University of Lodz, 12/16 Banacha Street, 90-237 Lodz, Poland; aleksandra.tonczyk@biol.uni.lodz.pl (A.T.); katarzyna.niedzialkowska@biol.uni.lodz.pl (K.N.); przemyslaw.bernat@biol.uni.lodz.pl (P.B.); 2 BioMedChem Doctoral School of University of Lodz and Lodz Institutes of Polish Academy of Sciences, 21/23 Matejki Street, 90-237 Lodz, Poland

**Keywords:** silver nanoparticles, mycogenic, synergism, ciprofloxacin, gentamicin, lipid and fatty acid profiles, antibiofilm

## Abstract

Silver nanoparticles (AgNPs) are used in a variety of different fields due to their excellent antimicrobial potential. Despite clear advantages, concerns about their toxicity have arisen, also concerning biogenic nanoparticles. Simultaneously, global healthcare is facing a problem of spreading antimicrobial resistance towards existing antibiotics. Using combined therapies involving AgNPs and antibiotics seems to be a promising solution to the above problems. The aim of this study was to evaluate the enhancement of the effectiveness of AgNPs, ciprofloxacin, and gentamicin against *Staphylococcus aureus* and *Pseudomonas aeruginosa*. The research involved the assessment of antimicrobial and antibiofilm-forming activities and the analysis of phospholipid and fatty acid profiles. Our results showed that combining the tested antimicrobials can enhance their activity against the tested bacterial strains. However, no effect was observed while mixing AgNPs with ciprofloxacin against *P. aeruginosa*. The most significant effect was obtained by combining 3.125 µg/mL of AgNPs with 0.125 µg/mL of gentamicin against *S. aureus*. It was also shown that the tested antimicrobials applied in combination exhibited an increased inhibitory activity towards bacterial biofilm formation by *S. aureus*. Lipidomic analysis revealed that under the influence of the tested antimicrobials, the properties of the cell membrane were altered in different ways depending on the bacterial strain.

## 1. Introduction

Silver nanoparticles (AgNPs) are one of the most commercialized and broadly used nanomaterials. This is because their high antimicrobial potential is considered as the strongest among metallic nanoparticles [1,2]. The complex mechanism of action involving a simultaneous impact on several cellular compartments and processes makes AgNPs effective against a broad spectrum of microorganisms. This property is especially interesting from the perspective of combating polymicrobial infections. It has also been noted that AgNPs are able to inhibit the ability of bacteria to adhere to surfaces and form biofilm [3,4]. All these features make it possible to utilize AgNPs in various industries, such as the food, textile, and cosmetics industries [5,6]. Moreover, AgNPs are used in biomedicine as medical device coatings or additives in medical formulations [7]. Despite the clear advantages of AgNPs, there are concerns regarding their potential cytotoxicity, especially in the case of long-term exposure to active concentrations [4]. It has been proven that AgNPs of biological origin show cytotoxic effects towards healthy mammalian cells [8,9,10]. Therefore, the common use of AgNPs as an antimicrobial agent has become questionable and the search for methods of lowering active doses is ongoing. One of the possible solutions is to combine two or more antimicrobial agents, acting in a synergistic way, in order to reduce dosages and minimize the toxicological effect of the mixture’s components [4].

At the same time, global healthcare is facing the problem of constantly growing antimicrobial resistance, resulting in both a high death rate among patients and the incrased expense of hospitalization caused by prolonged treatment [2]. With the still ongoing overuse of antibiotics not only in the field of medicine, but also in other branches, such as genetic engineering, animal breeding, and crop cultivation, the problem of the constant release of antibiotic residues into the natural environment has become inevitable. Concentrations of such compounds found in soil or water ecosystems vary between a few and hundreds of nanograms per liter or kilogram of soil. Additionally, despite the continuous introduction of antibiotics into the environment, their high persistence can enhance the threat posed [11,12]. Environmental antibiotic presence can force bacterial strains inhabiting polluted niches to develop resistance mechanisms, therefore promoting the occurrence of antimicrobial resistance [13]. The decreasing susceptibility of microorganisms to the existing conventional antibiotics and the emergence of multidrug-resistant (MDR) strains highlight the necessity of developing new, effective ways of treatment. Combined therapies using two or more agents with antimicrobial potential are a promising solution. It was found that AgNPs used simultaneously with antibiotics enhance the activity of various drugs, such as amoxicillin, vancomycin, tetracycline, neomycin, and chloramphenicol [1,3,4]. Moreover, this approach can lead to decreases in the quantity of the antibiotics required, as mixing AgNPs with antibiotics possibly reduces their required active dose [14], which, alongside the developing methods of antibiotic residue degradation [12], could reduce the threat posed to the natural environment by antibiotic pollution.

*Pseudomonas aeruginosa* and *Staphylococcus aureus* belong to the group of the most widespread human pathogens exhibiting antimicrobial resistance. These strains are among six constituting the ESKAPE group, associated with nosocomial infections, and are known to possess the ability of biofilm formation [15,16,17,18]. Bacterial biofilms can cover the surfaces of medical devices, such as catheters, and cause persistent infections, as cells forming biofilms can exhibit as much as 1000 times higher resistance to conventional antimicrobials compared to planktonic forms [19]. AgNPs have been proven to prevent biofilm formation and to successfully damage the developed biofilms [20].

Our previous study showed that *Gloeophyllum striatum*-derived AgNPs were active towards a broad spectrum of bacterial strains, including *S. aureus* and *P. aeruginosa*. It was also proven that they were able to inhibit biofilm formation by *P. aeruginosa*. However, the effective concentrations of the tested AgNPs showed increased toxicity towards the human fibroblast cell line [8]. These results emphasized the need for further research to seek possible ways of lowering active concentrations of AgNPs and maintaining their effectiveness.

The aim of this research was to evaluate the synergistic activity of AgNPs of mycological origin and two conventional antibiotics (ciprofloxacin and gentamicin) towards the Gram-positive bacterial strain *Staphylococcus aureus* ATCC 29213 and the Gram-negative bacterial strain *Pseudomonas aeruginosa* ATCC 27853. Both selected antibiotics are commonly used for the treatment of bacterial infections. Ciprofloxacin is a quinolone antibiotic displaying broad-spectrum activity based on the inhibition of DNA gyrase in bacterial cells leading to interference in the formation of double-stranded DNA. Ciprofloxacin is known to affect several Gram-positive bacterial strains, for example *Staphylococcus,* as well as Gram-negative strains, including *P. aeruginosa*. Gentamicin belongs to aminoglycoside family of antibiotics and is active against Gram-negative bacteria by inhibiting the synthesis of bacterial proteins. In both cases, the increasing resistance of bacterial strains, including *S. aureus* and *P. aeruginosa*, to antibiotic action has been reported [21,22]. Consequently, an investigation concerning the synergistic effect of these two antibiotics with AgNPs can shed light on possible solutions to the still-developing antimicrobial resistance problem. The assessment included the examination of antibacterial activity, the evaluation of the inhibitory effect of the tested antimicrobials on biofilm formation, and the detection of changes in the phospholipid and fatty acid compositions caused by AgNPs and antibiotics applied in selected concentrations. The lipidomic analysis is vital in the investigation of AgNPs’ influence on bacterial cells, as the first step of the antimicrobial action of AgNPs is the interaction with cell envelope’s adhesion to the cell wall and/or cell membrane. This can lead to the destabilization of the cell membrane structure and its properties, such as permeability and fluidity [23,24]. The changes detected in lipid profiles in bacterial cells treated with AgNPs or a mixture of nanoparticles and selected antibiotics can provide information about possible shifts in AgNPs’ action when combined with antibiotics with known modes of antibacterial activity.

## 2. Results

### 2.1. Antimicrobial Activity of AgNPs, Antibiotics, or a Combination of the Agents

**Figure 1:**
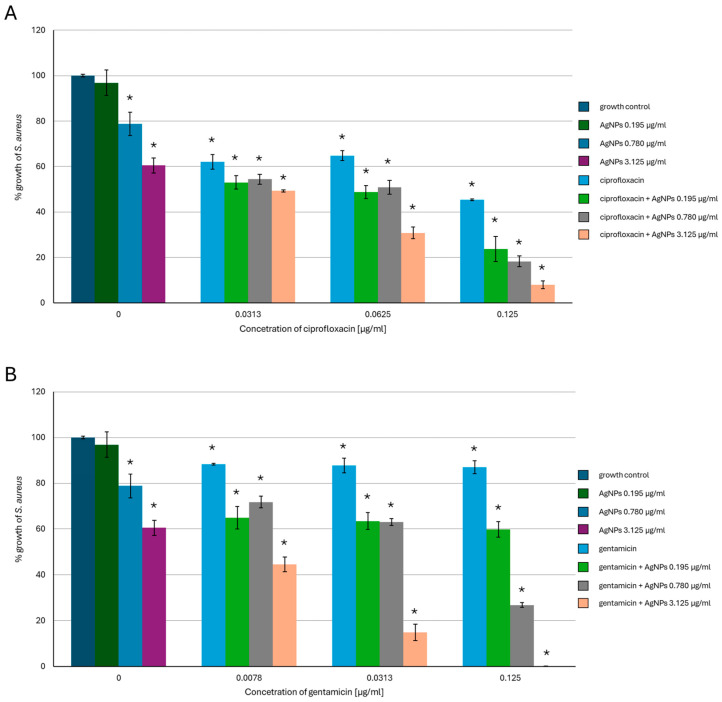
Antibacterial activity of mycogenic silver nanoparticles (AgNPs), ciprofloxacin (**A**), and gentamicin (**B**) against *S. aureus*. The results are shown as average percentage values with standard deviations of optical density (OD) of the biotic control. The statistical significance is shown by an asterisk (* *p* < 0.05).

Our results showed that the simultaneous use of *G. striatum*-derived AgNPs (from now on described as ‘AgNPs’ throughout this section) and the tested antibiotics enhanced the activity of the nanoparticles against *S. aureus* and *P. aeruginosa*. In *S. aureus* (Figure 1), the AgNPs used alone at the concentrations of 0.195 µg/mL, 0.78 µg/mL, and 3.125 µg/mL caused about 5%, 20%, and 40% inhibition of bacterial growth compared to growth control samples, respectively. Ciprofloxacin used alone against *S. aureus* caused about 40% bacterial growth inhibition at the concentrations of 0.0313 µg/mL and 0.0625 µg/mL and 55% growth inhibition at the concentration of 0.125 µg/mL. Mixing AgNPs in all concentrations with 0.125 µg/mL of ciprofloxacin resulted in an over two-fold enhancement of the AgNPs’ activity, which reached a 95% growth-inhibiting effect in the case of the highest concentration of the nanoparticles tested. Gentamicin used alone was less harmful to *S. aureus* compared to fluoroquinolone. However, combining gentamicin at a concentration of 0.125 µg/mL with 3.125 µg/mL of AgNPs caused a complete inhibitory effect on the growth of the tested bacterial strain and an about 90% inhibitory effect when the concentration of the antibiotic was four times lower.

**Figure 2:**
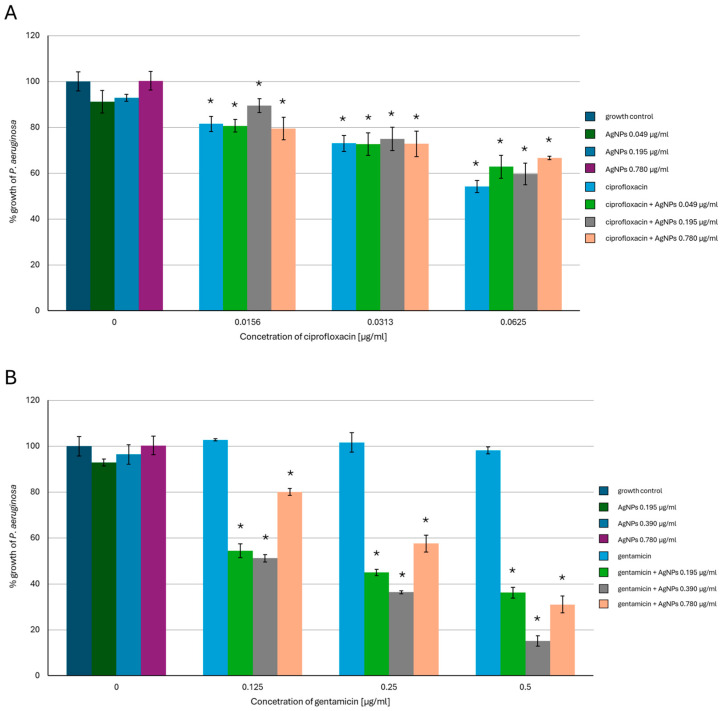
Antibacterial activity of mycogenic AgNPs, ciprofloxacin (**A**), and gentamicin (**B**) against *P. aeruginosa*. The results are shown as average percentage values with standard deviations of optical density (OD) of the biotic control. The statistical significance is shown by an asterisk (* *p* < 0.05).

In *P. aeruginosa* (Figure 2), AgNPs in the concentration range of 0.049–0.78 µg/mL caused a slight inhibition of bacterial growth. Ciprofloxacin used alone at the concentrations of 0.0156 µg/mL and 0.0313 µg/mL inhibited bacterial growth by about 20% and by about 50% when applied at the highest concentration tested. Mixing each concentration of AgNPs with 0.0156 µg/mL of ciprofloxacin resulted in a slight enhancement of the AgNPs’ activity against *P. aeruginosa*. When combined with higher antibiotic concentrations, the AgNPs’ activity was enhanced and caused bacterial growth inhibition by up to 40%. However, there were no vivid differences between the effectiveness of the tested AgNP concentrations. Gentamicin used alone was less effective against *P. aeruginosa* compared to the other antibiotics, and no inhibitory effect was observed for all tested concentrations. Mixing 0.195 µg/mL or 0.390 µg/mL of AgNPs with each tested concentration of gentamicin enhanced the effectiveness of AgNPs compared to when AgNPs were used alone. When 0.39 µg/mL of AgNPs and the highest gentamicin concentration were used, the effectiveness increased by 75%. Interestingly, the effect of increasing the effectiveness of antimicrobials applied simultaneously was weaker in the case of the highest concentration of AgNPs tested mixed with each variant of gentamicin used.

### 2.2. Antibiofilm-Forming Activity of AgNPs, the Antibiotics and a Combination of the Agents

**Figure 3:**
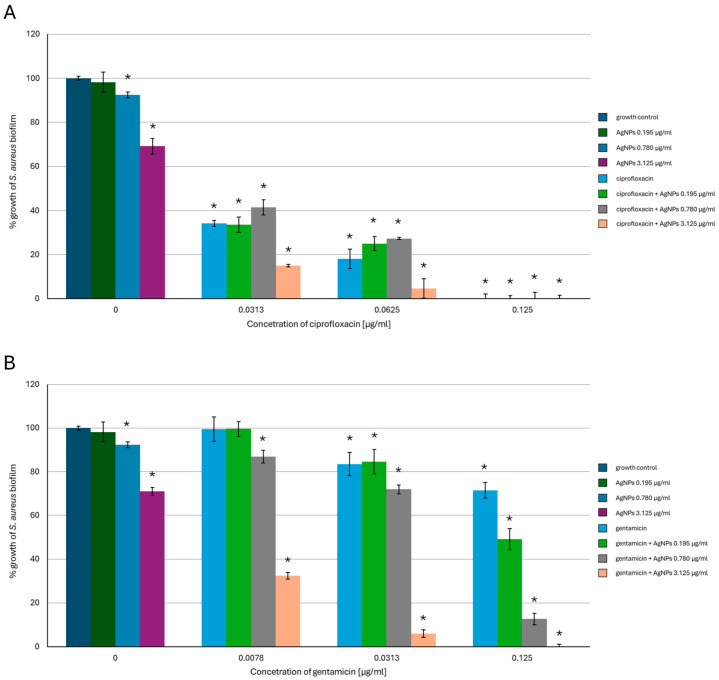
Antibiofilm-forming activity of mycogenic AgNPs, ciprofloxacin (**A**), and gentamicin (**B**) against *S. aureus*. The results are shown as average percentage values with standard deviations of optical density (OD) of the biotic control. The statistical significance is shown by an asterisk (* *p* < 0.05).

The results of the evaluation of the antibiofilm-forming activity revealed that combining AgNPs with ciprofloxacin or gentamicin enhanced the inhibitory effect of the tested antimicrobials towards bacterial biofilm. It was shown that, in the case of *S. aureus* (Figure 3), mixing the lowest tested concentration of AgNPs with 0.0313 µg/mL of ciprofloxacin enhanced the antibiofilm activity of AgNPs by 50%. The same concentration of the antibiotic combined with 3.125 µg/mL of AgNPs caused a reduction in biofilm formation of over 20% compared to the biotic control, while a two-fold higher ciprofloxacin concentration combined with the same AgNPs variant caused over 90% inhibition of *S. aureus* biofilm-forming activity. Additionally, 0.125 µg/mL of ciprofloxacin alone or combined with all tested AgNP concentrations completely inhibited biofilm formation. In the case of gentamicin, mixing each antibiotic variant with the highest AgNP concentration enhanced the antibiofilm activity. Furthermore, 3.125 µg/mL of AgNPs combined with 0.0078 µg/mL of gentamicin caused about 70% inhibition of *S. aureus* biofilm formation and over 90% inhibition when combined with the antibiotic at a concentration of 0.0313 µg/mL. Mixing 0.125 µg/mL of gentamicin with 0.78 µg/mL of AgNPs reduced bacterial biofilm formation by about 90%, while the same concentration of the antibiotic combined with 3.125 µg/mL of AgNPs caused a complete inhibition of biofilm formation.

**Figure 4:**
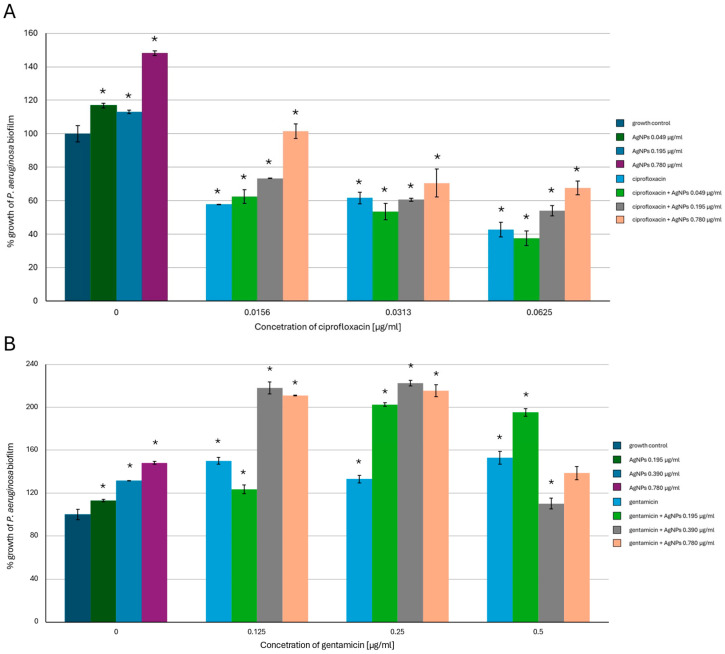
Antibiofilm-forming activity of mycogenic AgNPs, ciprofloxacin (**A**), and gentamicin (**B**) against *P. aeruginosa*. The results are shown as average percentage values with standard deviations of optical density (OD) of the biotic control. The statistical significance is shown by an asterisk (* *p* < 0.05).

An inhibitory effect on *P. aeruginosa* biofilm formation (Figure 4) of the tested antimicrobials was also detected; however, the addition of AgNPs alone caused an increase in the biofilm-forming activity of this bacterial strain. The best inhibitory result was observed in the case of mixing 0.049 µg/mL of AgNPs with 0.0625 µg/mL of ciprofloxacin. Here, the growth of bacterial biofilm was reduced to less than 40% of the biotic control. When AgNPs were combined with gentamicin, the stimulation of *P. aeruginosa* biofilm-forming activity was observed. This phenomenon reached the highest value of more than 200% of the biotic control in the cases of 0.39 µg/mL and 0.78 µg/mL of AgNPs combined with 0.125 µg/mL and 0.25 µg/mL of gentamicin, respectively. There was no inhibitory effect on the biofilm-forming activity compared to the biotic control in this variant of the experiment.

### 2.3. Changes in the Phospholipid Profiles of the Tested Bacteria in the Presence of AgNPs, the Antibiotics or a Combination of Agents

In our research, four different species of phospholipids (PL) were detected in the tested bacterial species, namely phosphatidylethanolamines (PE), phosphatidylglycerols (PG), and lysyl-phosphatidylglycerols (L-PG) in *S. aureus* (Table 1), and phosphatidylcholines (PC), PE, and PG in *P. aeruginosa* (Table 2). In *S. aureus*, the most numerous class of phospholipids was L-PG, reaching 67.45% of the total phospholipid content in the growth control. The content distribution of the remaining PL species in biotic samples comprised 31.77% and 0.79% for PG and PE, respectively. The addition of the tested antimicrobials caused a decrease in the amount of PE, and the most significant change was observed in the case of 3.125 µg/mL of AgNPs and 0.0078 µg/mL of gentamicin mixture. In the case of PG, the addition of AgNPs, gentamicin, and AgNPs mixed with ciprofloxacin caused decreases in the phospholipid contents. Here, the % amount of PG in the presence of AgNPs was two times lower compared to the biotic control. The presence of gentamicin caused a decrease in the content of PG by about 20%, while the mixture of nanoparticles and ciprofloxacin caused a 30% decrease in PG content. On the other hand, the content of L-PG increased in the presence of the same antimicrobials. The most visible change was caused by the presence of AgNPs alone—the content of L-PG increased to 83.4%. In *P. aeruginosa*, the most numerous phospholipids species was PG, with a content of 71.18% in the biotic controls. The contents of the remaining phospholipid fractions were 26.47% for PE and 2.35% for PC. The addition of 0.78 µg/mL of AgNPs alone did not influence the content of PC, but it caused a slight decrease in PG and an increase in PE quantities. Both antibiotics alone caused a decrease in the amount of PC by about 30%, while the addition of the AgNPs and gentamicin mixture lowered the PC content twofold. The same effect of AgNPs combined with gentamicin was observed in PG, as the content of this phospholipid species decreased to 65.18%. The content of PE was increased by the presence of AgNPs and ciprofloxacin alone and by AgNPs combined with gentamicin, where the change was the most visible with the PE content, which increased by 30% compared to the growth control.

### 2.4. Changes in the Fatty Acid Content in the Tested Bacterial Strains in the Presence of AgNPs, the Antibiotics or a Combination of the Agents

The results obtained for *S. aureus* (Table 3) revealed the presence of 14 types of fatty acids in the samples. The most numerous fractions in the biotic control were a-15:0 and 18:0 with the contents of 40.98% and 15.68%, respectively. The least numerous fractions were 15:0 and 14:0, where the percentage content was below 1%. The most visible changes in the content of fatty acids caused by the presence of the tested antimicrobials were observed in the case of 18:0. Here, the presence of AgNPs and antibiotics alone or in combination caused an increase in the quantity of this fatty acid type and gentamicin used alone showed the most significant effect. On the other hand, the tested antimicrobials caused a decrease in the content of a-15:0 fatty acid, and again gentamicin exhibited the strongest effect.

The investigation of the fatty acid profile of *P. aeruginosa* resulted in the detection of nine fatty acid species (Table 4). Here, the most numerous classes were 16:0 and 18:1, with percentage contents of 40.84% and 36.19%, respectively. The least numerous groups were the i-12:0 and 8:0 fatty acids. The most visible effect of the tested antimicrobials on the content of fatty acids was observed in the 16:1 species. Here, in the presence of AgNPs combined with gentamicin, the content of fatty acids increased by about 17% compared to the biotic control.

## 3. Discussion

AgNPs are one of the most investigated metallic nanoparticles and have gained significant attention not only in the field of biomedicine but also in other industries. One of the most crucial properties of AgNPs is their strong inhibitory activity against a broad spectrum of microorganisms. The use of AgNPs is, therefore, considered as a potentially promising substitute for conventional antibiotic therapy [6,7]. The latter option outweighs the conventional routes, for example by providing reducing, stabilizing, and capping agents in the form of metabolites characteristic for the organism species used in the process. Mycogenic AgNPs are proven to be coated with the protein layer which can positively affect the functionality of nanoparticles [25]. However, there are still unresolved doubts concerning the potential toxicity of AgNPs. It has been reported that AgNPs can damage mammalian cells via the impairment of mitochondrial functionality, increases in cell membrane permeability, or the generation of reactive oxygen species [4,26]. Additionally, the still ongoing emergence of antimicrobial resistance towards available therapeutics has become a worldwide challenge. One of the proposed solutions involves combining nanoparticles with drugs [27]. AgNPs have been proven to enhance the activity of various antibiotics against different groups of microorganisms either in a synergistic or additive manner. According to the above, it seems possible to reduce the need for high antibiotic concentrations while lowering the effective dosages of AgNPs [4,28].

In our previous study, the antimicrobial activity of mycogenic AgNPs synthesized using *G. striatum* post-culture liquid was evaluated. It was found that AgNPs synthesized in the conditions of 4 °C without shaking were the most suitable for further investigation. The established minimal inhibitory concentration (MIC) values of this particular AgNP type were 12.5 µg/mL and 1.56 µg/mL for *S. aureus* and *P. aeruginosa*, respectively. Interestingly, these AgNPs did not cause a severe cytotoxic effect on the tested human fibroblast cell line in the concentration corresponding to the MIC value in *P. aeruginosa* [8]. The present research showed that combining *G. striatum*-derived AgNPs with ciprofloxacin or gentamicin enhanced the activity of the tested antimicrobials against *S. aureus* and *P. aeruginosa* compared to their effectiveness when used alone. For example, 3.125 µg/mL of AgNPs combined with 0.125 µg/mL of gentamicin completely inhibited the growth of *S. aureus*, thus reducing the effective dose of AgNPs by four times. In *P. aeruginosa*, 0.39 µg/mL of AgNPs mixed with 0.5 µg/mL of gentamicin caused almost 90% inhibition of bacterial growth compared to the biotic control. Wang et al. [29] showed that the activity of chemically synthesized AgNPs against *S. aureus* was also enhanced in the presence of gentamicin. They concluded that a possible explanation for this phenomenon could be the increased dissolution of AgNPs or the facilitated interactions of nanoparticles with the cell surface in the presence of the antibiotic. Combining AgNPs with ciprofloxacin was most effective in the case of *S. aureus*. Here, the activity of AgNPs at a concentration of 3.125 µg/mL was increased twofold when mixed with 0.125 µg/mL of antibiotic compared to the effectiveness of AgNPs used alone. The synergistic effect between biogenic AgNPs and ciprofloxacin against *S. aureus* was proven by Bhat et al. Using the disc diffusion method, they observed that combining a 5 µg/disc of the antibiotic and a 20 µg/disc of AgNPs caused the zones of growth inhibition to increase two times when compared to the effect caused by AgNPs acting alone [30]. When it came to *P. aeruginosa*, only a slight enhancement of AgNP activity in the presence of ciprofloxacin was observed, as 0.0313 µg/mL and 0.0625 µg/mL of the antibiotic mixed with each tested concentration of AgNPs enhanced their effectiveness by about 40% and no differences in the effectiveness of various AgNP concentrations were observed. Our findings are in opposition to the results of Nikparast and Saliani [31]. In their study, the potential synergistic effect of plant-derived AgNPs originating from *Amaranthus retroflexus* combined with ciprofloxacin was evaluated towards the human pathogenic bacteria *P. aeruginosa* and plant pathogenic bacteria *P. syringae*. They found that in the presence of 6.25, 12.5, and 25 μg/mL of AgNPs, the MIC value of ciprofloxacin was reduced from 0.125 μg/mL to 0.0625 μg/mL in the case of *P. aeruginosa* and from 0.25 μg/mL to 0.0625 μg/mL in *P. syringae*. Based on their results, it can be concluded that *G. striatum*-derived AgNPs used alone showed 16 times better efficacy against *P. aeruginosa* compared to nanoparticles synthesized using *A. retroflexus* leaf extract [8,31].

Microbial biofilms are communities of microorganisms that possess the ability to adhere to different surfaces. Bacteria forming biofilms are surrounded by an extracellular matrix consisting of self-secreted compounds. This envelope forms a hospitable environment and protects cells from external stresses, such as antimicrobial agents [32,33]. Antimicrobial resistance can be as much as 1000 times stronger in biofilm forms compared to in planktonic bacteria and, as such, is one of the major concerns in contemporary healthcare [34,35]. It has been reported that AgNPs are able to successfully suppress biofilm formation [36]. In our study, the antibiofilm activity of AgNPs, ciprofloxacin, gentamicin, or a combination of the agents was evaluated against *S. aureus* and *P. aeruginosa*. The *S. aureus* strain used in our study was classified as a weak biofilm former [37]. The *P. aeruginosa* strain had been previously described as a strong biofilm former [8]. It was found that combining AgNPs with both tested antibiotics was effective against *S. aureus* and that the inhibitory activity towards bacterial biofilm of the tested antimicrobials was enhanced. Our results revealed that, in the case of *P. aeruginosa*, the inhibitory effect on biofilm production caused by the tested antimicrobials was weaker. When mixed with 0.0625 µg/mL of ciprofloxacin, 0.049 µg/mL of AgNPs caused biofilm reduction of about 60% compared to the biotic control. In the case of mixing AgNPs with gentamicin, no inhibitory effect was observed. We discovered, however, that AgNPs in the tested concentrations caused a stimulatory effect on biofilm formation, and the same phenomenon was observed when AgNPs were used in combination with gentamicin. According to our previous study, the AgNP concentration of 0.78 µg/mL had a sublethal effect on *P. aeruginosa*, as the established MIC value was equal to 1.56 µg/mL [8]. Therefore, it can be concluded that the concentrations of AgNPs that do not inhibit bacterial growth completely can increase biofilm formation by *P. aeruginosa*. Similar results were obtained by Yu and Alvarez, who found that a mixed microbial culture from a wastewater treatment plant after exposition to sublethal concentrations of AgNPs showed enhancement in the biofilm formation of up to six times compared to the biotic control. The same effect was observed in their study on the *P. aeruginosa* PAO1 strain. The authors suggested that the promotion of biofilm formation could be a bacterial defense mechanism against AgNPs’ toxicity [38]. Moreover, in the study cinducted by Kumar et al. (2024) it was found that sub-MIC concentrations of gentamicin led to an increase in biofilm formation in two environmental isolates of *P. aeruginosa*. Their research showed that during antibiotic treatment, the contents of the biofilm-forming components, such as exoproteins, eDNA, exolipids, and exopolymeric substances, were higher than in the control samples without the addition of gentamicin. Therefore, it can be concluded that, under the stress of antimicrobial treatment, *P. aeruginosa* is able to develop additional defense mechanisms, leading to increased biofilm formation [39]. Contrastingly, it was also stated in the other study that the effect of sub-MIC concentrations of AgNPs enhancing biofilm-forming activity was not detected in *S. aureus* [40]. On the other hand, in another study, it was reported that the presence of ciprofloxacin could stimulate the production of bacterial biofilm in *S. aureus* [41]. Nevertheless, this phenomenon was not observed in our study, neither when ciprofloxacin was applied alone nor in combination with nanoparticles.

The bacterial cell membrane is mainly composed of phospholipids, whose major function is the formation of a semipermeable barrier. Phospholipids are also involved in some cellular processes. One of the most important tasks fulfilled by membrane phospholipids is adaptation to inhospitable environment conditions. Thus, changes in the membrane phospholipids’ content and composition can be an important factor of environmental stress [42,43]. In *S. aureus*, polar phosphatidylglycerol (PG) and lysyl-phosphatidylglycerols (L-PG) are among the most numerous fractions of lipids forming cell membranes. An increased L-PG content shows a protective effect, as the modification of PG with lysine reduces the negative charge of the membrane. This effect leads to the increased electrostatic repulsion of positively charged extracellular compounds [42]. Our results showed that the amount of L-PG increased when AgNPs, gentamicin, or a combination of AgNPs and ciprofloxacin were used. Thus, the occurrence of the process of adaptation to suboptimal growth conditions in *S. aureus* was revealed. In *P. aeruginosa*, commonly present groups of phospholipids are PG and phosphatidylethanolamines (PE). PE and PG constitute the majority of phospholipids forming the cell membrane [44]. Our results regarding the composition of *P. aeruginosa* membrane phospholipids are in agreement with the above data. Fatty acids determine the biophysical properties of cell membranes. In *S. aureus*, bacterial membranes are formed by straight-chained and branch-chained saturated fatty acids [45,46]. This phenomenon was confirmed by our study, where all of the detected *S. aureus* fatty acids were saturated. Branch-chained acids accounted for about 71% of all fatty acids in growth controls, while straight-chained acids constituted the remaining 29%. Straight-chained acids form a thick bilayer with low permeability, while branch-chained fatty acids, including iso- and anteiso- methyl forms, enhance the fluidity of the membrane [46]. In *P. aeruginosa*, unsaturated and saturated straight-chained fatty acids were detected, alongside branch-chained ones. The difference between the Gram-positive and Gram-negative bacterial strains found in our study is in the agreement with the available literature data. Dubois-Brisonnet et al. revealed that in planktonic forms of *S. aureus*, the iso-branch-chained fatty acids and anteiso-branch-chained fatty acids classes constituted about 70% of all fatty acids detected. There were no unsaturated fatty acids found. These data agree with the results obtained in our study. In the same study, unsaturated fatty acids were detected in *P. aeruginosa*. However, these species constituted more than a half of the whole fatty acid content, which is in slight disagreement with our results [47].

Our research revealed that changes in the contents of fatty acid species in the presence of AgNPs and antibiotics were different among the tested bacterial strains. In *S. aureus*,the two most numerous fatty acid species, octadecanoic acid (18:0) and iso-pentadecanoic acid, changed in the presence of all of the tested antimicrobials alone or in combination. The content of straight-chained acid increased by as much as 80% compared to the biotic control in the case of gentamicin used alone. Contrastingly, the amount of branch-chained fatty acid decreased by more than 15% in the same samples. Similar changes were also observed in less numerous fatty acid species. These results indicated that the presence of the tested antimicrobials promoted changes in the bacterial membrane composition, resulting in decreased permeability and fluidity. Indeed, changing the properties of the cell membrane into a more solid state is considered to be a resistance response of *S. aureus* to antibiotic stress [48]. The results regarding *P. aeruginosa* fatty acid composition showed that saturated acids constituted up to 55% of all species, while unsaturated and branch-chained acids accounted for the remaining amount, while the branch-chained acids were present only in trace quantities in the biotic controls. The addition of AgNPs, ciprofloxacin, gentamicin, or a combination of the antimicrobials caused changes in the amounts of the second and third most numerous fatty acid fractions, namely saturated octadecanoic acid (18:0) and unsaturated oleic acid (18:1), respectively. The content of saturated acids decreased in the presence of every antimicrobial used except for ciprofloxacin applied alone. Contrastingly, the amount of oleic acid increased in the same cases. An increased content of unsaturated fatty acids was also observed in the case of palmitoleic acid (16:1) in *P. aeruginosa* samples incubated with AgNPs alone, AgNPs mixed with ciprofloxacin and AgNPs mixed with gentamicin. The changes in the saturated and unsaturated fatty acid contents are a primary mechanism of Gram-negative bacteria to regulate cell membrane fluidity [48]. Based on our results, it can, therefore, be concluded that the tested antimicrobials enhanced fluidity of the *P. aeruginosa* cell membrane.

The fatty acid profile structure can be a factor impacting biofilm-forming activity. In general, bacterial cells forming biofilm contain higher amounts of saturated acids compared to planktonic cells [47,49]. It has been found that lower membrane fluidity enhances biofilm-forming activity, which can be considered as a stress response [50,51]. However, our results on *P. aeruginosa* showed an increase in cell membrane fluidity in the presence of AgNPs and the tested antibiotics. It is speculated that this phenomenon can negatively impact the development of biofilm by disturbing the stability of the biofilm form during the formation process [52].

## 4. Materials and Methods

### 4.1. Materials

The tested bacterial strains were purchased from the American Type Culture Collection (ATCC, USA). AgNPs of mycological origin were obtained with the use of *Gloeophyllum striatum* DSM 9592 post-culture liquid in the manner described previously [8]. In this study, nanoparticles synthesized at 4 °C without shaking were used. The tested antibiotics, namely ciprofloxacin and gentamicin, were purchased from Merck (Darmstadt, Germany). The Mueller–Hinton broth (BBL^TM^) medium was obtained from Becton Dickinson (Warsaw, Poland). Acetic acid and cristal violet came from Chempur (Piekary Slaskie, Poland).

### 4.2. Antimicrobial Activity of AgNPs, Antibiotics and Agents Combined

The assessment of the antimicrobial activity of AgNPs, ciprofloxacin, gentamicin, or a combination of the agents was performed using the microdilution method according to the Clinical and Standard Laboratory Institute (CSLI) guidelines M07 (11th Edition) towards two reference bacterial strains, namely Gram-positive *S. aureus* ATCC 29213 and Gram-negative *P. aeruginosa* ATCC 27853. The differences in growth of the tested bacterial strains in the presence of selected antimicrobial agents were examined in 96-well cell culture plates in Mueller–Hinton broth medium. The final concentration range of the tested antibiotics was 0–8 µg/mL in both experimental variants and the final concentration ranges of AgNPs were 0–12.5 µg/mL and 0–1.56 µg/mL for *S. aureus* and *P. aeruginosa*, respectively. A bacterial inoculum was prepared in Mueller–Hinton broth medium and added to each cell, where the final bacterial density reached 5 × 105 colony-forming units (CFU)/mL. Adequate abiotic controls were also prepared. All the samples and abiotic controls were then incubated for 24 h at 37 °C. After incubation, the OD was measured at a wavelength of 630 nm using a MultiskanTM FC Microplate Photometer (Thermo Fisher Scientific, Pudong, Shanghai, China). Based on the primary results, the selected variants were further analyzed. The selected concentrations of the tested antimicrobials were distributed as follows: 0.195, 0.78, and 3.125 µg/mL of AgNPs, 0.0313, 0.0625, and 0.125 µg/mL of ciprofloxacin and gentamicin in the case of *S. aureus*, and 0.049, 0.195, 0.39, and 0.78 µg/mL of AgNPs, 0.0156, 0.0313, and 0.0625 µg/mL of ciprofloxacin, and 0.125, 0.25, and 0.5 µg/mL of gentamicin in the case of *P. aeruginosa*.

### 4.3. Antibiofilm-Forming Activity of AgNPs, Antibiotics and Agents Combined

The ability of the tested antimicrobials to inhibit bacterial biofilm formation by *S. aureus* and *P. aeruginosa* was determined in the same experiment schemes as described in the previous paragraph. The assessment of changes in the biofilm formation was performed with the use of crystal violet solution according to the method described in our previous research [8].

### 4.4. Changes in the Phospholipid Profiles and Fatty Acid Contents in the Tested Bacteria in the Presence of AgNPs, the Antibiotics, or a Combination of the Agents

The phospholipid and fatty acid profile analysis was performed in the selected concentration of antibiotics and AgNPs for both tested bacterial strains. For *S. aureus*, the concentration of AgNPs was 3.125 µg/mL and the concentrations of antibiotics were 0.0625 µg/mL and 0.0078 µg/mL for ciprofloxacin and gentamicin, respectively. For *P. aeruginosa*, the tested AgNP concentration was equal to 0.78 µg/mL, while the concentrations of antibiotics were 0.0313 µg/mL and 0.125 µg/mL for ciprofloxacin and gentamicin, respectively. All tested samples and biotic and abiotic controls were prepared in Mueller–Hinton broth medium and incubated for 24 h at 37 °C. After the incubation period, the tested samples and biotic controls were transferred to 50 mL falcon tubes and centrifuged at 20 °C/8000 rpm/6 min. Then, the supernatant was removed, and 100 mg of obtained biomass from each sample was placed in 2 mL Eppendorf tubes. Every tube was then filled with 1 mL of methanol and glass pearls and homogenized on a Fast-Prep-24 h Instrument (MP Biomedicals, Eschwege, Germany). When the process ended, all the samples were again centrifuged for 4 min/7000 rpm at room temperature and the methanolic phases were transferred to new tubes.

Phospholipid analysis was carried out using LC-MS/MS (ExionLC AC UHPLC system (Sciex, Framingham, MA, USA)) with a 4500 Q-TRAP mass spectrometer (Sciex, USA). The obtained lipid extract was first fractionated using the UHPLC system. For this, 10 μL of the extract was injected into a Kinetex C18 column (50 mm × 2.1 mm, particle size: 5 μm; Phenomenex, Torrance, CA, USA) at a flow rate of 500 µL min^−1^ [53]. The column temperature was maintained at 40 °C. The mobile phases used were water (A) and methanol (B), both of which were supplemented with 5 mM ammonium formate. The solvent elution started from 70% B and was then increased to 95% B over 1.25 min and maintained at 95% B for 6 min before returning to 70% B over 3 min. The mass spectrometric analysis was carried out using a mass spectrometer equipped with an electrospray ionization (ESI) source, under the following conditions: spray voltage −4500 V, curtain gas 25 psi, nebulizer gas 50 psi, turbo gas 60 psi, and ion source temperature 600 °C. Data analysis was performed using the Analyst™ v1.6.3 software (Sciex, USA). The qualitative and quantitative analyses were carried out according to the methods described previously [53] with the use of multiple reaction monitoring (MRM) pairs.

Fatty acids were analyzed according to the method described earlier [54]. A lipid sample was diluted in 1.5 mL methanol and transferred to a screw-capped glass test tube. To this lipid solution, 0.2 mL toluene and 0.3 mL HCl solution (8.0%) were added [55]. The tube was vortexed and then incubated overnight at 45 °C. After cooling to room temperature, 1 mL hexane and 1 mL water (deionized) were added for the extraction of fatty acid methyl esters (FAMEs). The tube was vortexed, and 0.3 mL of the hexane layer was moved to the chromatographic vial. The FAMEs analysis was conducted with an Agilent Model 7890 gas chromatograph connected to a 5975C mass detector (Agilent, Santa Clara, CA, USA). Helium and a capillary column of HP 5 MS methyl polysiloxane (30 m × 0.25 mm i.d. × 0.25 mm ft) were applied. The temperature of the column was maintained at 60 °C for 3 min, then increased to 212 °C at a rate of 6 °C min^−1^, followed by an increase to 245 °C at a rate of 2 °C min^−1^, and, finally, to 280 °C at a rate of 20 °C min^−1^, at which it was held for 10 min. Split injection of the injection port at 250 °C was employed. Fungal fatty acids were identified by comparison with authenticated reference standards (Sigma, Darmstadt, Germany, Supelco, Darmstadt, Germany).

### 4.5. Statistical Analysis

All experiments presented in this study were performed in four replicates (*n* = 4). The results of the examination of phospholipid and fatty acid contents were analyzed using a one-way ANOVA test with * *p* < 0.05 in order to estimate the statistical significance. During the analysis of the results of antimicrobial and antibiofilm activity, a one-way ANOVA test was applied in the case of an antimicrobial agent used alone, and a two-way ANOVA test was employed for the evaluation of the results obtained when antimicrobials were combined. Both tests were performed with * *p* < 0.05. The estimations and all calculations were carried out by using Excel, Microsoft^®^ Office 2021 (Microsoft Corporation, Redmont, WA, USA). The results shown in the figures and tables are expressed as the average values with the standard deviation (SD).

## 5. Conclusions

Our research showed that the tested mycogenic AgNPs and the antibiotics ciprofloxacin and gentamicin can perform synergistic action towards two pathogenic bacterial strains, namely *S. aureus* and *P. aeruginosa*. It was also revealed that selected antimicrobials possessed antibiofilm-forming activity that was enhanced by a simultaneous application of AgNPs and an antibiotic in almost all variants of the experiment. However, a more significant increase in antimicrobial and antibiofilm-forming efficacy of the tested antimicrobials was detected in the case of *S. aureus*, where a total inhibition of bacterial growth and biofilm formation was reached. The analysis of phospholipid and fatty acid profiles revealed differences in the response to unhospitable growth conditions in the tested bacteria caused by the presence of AgNPs and antibiotics. More vivid changes in the cell membrane properties were observed again in *S. aureus*, alongside with the highest susceptibility to the tested antimicrobials. The observed effect of the tested antimicrobials towards *P. aeruginosa* did not lead to the complete inhibition of bacterial cell growth. Moreover, it was revealed that the sublethal concentrations of AgNPs and antibiotics stimulated bacterial biofilm formation by the tested strain. Additionally, the changes in the phospholipid and fatty acid contents in the case of *P. aeruginosa* only slightly indicated the enhancement of cell membrane fluidity. Given these facts, it can be concluded that in *P. aeruginosa*, an increased biofilm-forming activity is the first defense mechanisms to the action of the tested antimicrobials. Thus, combining AgNPs with the commonly used antibiotics ciprofloxacin and gentamicin can be an effective way to enhance their antimicrobial action and to lower the sufficient doses of the antimicrobials. However, the effectiveness of this method can differ among various bacterial strains, where an increase in the antimicrobial activity does not correlate with a complete inhibitory effect on bacterial growth. Moreover, the revealed stimulatory effect of AgNPs and their combinations with antibiotics on *P. aeruginosa* biofilm formation should attract more attention, as the mentioned phenomenon can pose an additional threat in the field of healthcare.

## Figures and Tables

**Table 1:** The influence of the tested antimicrobials dosed alone and in combination on the phospholipid profile of *S. aureus* ATCC 29213. Antimicrobials were used at the following concentrations: 3.125 µg/mL of silver nanoparticles (AgNPs), 0.0625 µg/mL of ciprofloxacin, and 0.0078 µg/mL of gentamicin. Asterisk * (*p* < 0.05) indicates values that differ significantly from the control.

Phospholipid Species	Growth Control	AgNPs	Ciprofloxacin	Gentamicin	AgNPs + C	AgNPs + G
PE 28:0	0.03 ± 0.028	0.01 ± 0.000	0.03 ± 0.007	0.02 ± 0.007	0.02 ± 0.007	0.01 ± 0.000
PE 32:2	0.24 ± 0.014	0.11 ± 0.007 *	0.36 ± 0.092	0.23 ± 0.134	0.3 ± 0.000 *	0.16 ± 0.198
PE 28:0	0.03 ± 0.028	0.01 ± 0.000	0.03 ± 0.007	0.02 ± 0.007	0.02 ± 0.007	0.01 ± 0.000
PE 30:0	0.02 ± 0.007	0.04 ± 0.035	0.02 ± 0.007	0.04 ± 0.035	0.01 ± 0.014	0.01 ± 0.007
PE 30:1	0.01 ± 0.000	0.01 ± 0.007	0.02 ± 0.000	0.01 ± 0.007	0.01 ± 0.007	0.03 ± 0.007
PE 31:0	0.03 ± 0.000	0.02 ± 0.007	0.04 ± 0.021	0.02 ± 0.007	0.04 ± 0.021	0.01 ± 0.000
PE 31:1	0.02 ± 0.007	0.02 ± 0.028	0.01 ± 0.000	0.02 ± 0.007	0.03 ± 0.014	0.02 ± 0.014
PE 32:0	0.15 ± 0.106	0.20 ± 0.226	0.06 ± 0.014	0.11 ± 0.042	0.05 ± 0.035	0.10 ± 0.049
PE 32:1	0.17 ± 0.007	0.10 ± 0.085	0.04 ± 0.028 *	0.02 ± 0.000 *	0.04 ± 0.021 *	0.03 ± 0.000 *
PE 32:2	0.24 ± 0.014	0.11 ± 0.007 *	0.36 ± 0.092	0.23 ± 0.134	0.3 ± 0.000 *	0.16 ± 0.198
PE 33:2	0.13 ± 0.007	0.09 ± 0.099	0.02 ± 0.007 *	0.02 ± 0.007 *	0.03 ± 0.007 *	0.00 ± 0.000 *
PG 30:1	4.76 ± 0.06	1.93 ± 0.219 *	5.37 ± 0.516	2.80 ± 0.184 *	3.20 ± 1.195	3.85 ± 2.171
PG 31:1	17.57 ± 2.638	8.48 ± 0.976	19.43 ± 2.857	13.02 ± 3.330	11.15 ± 1.853	18.34 ± 6.824
PG 32:0	6.36 ± 1.513	3.59 ± 0.042	5.90 ± 1.584	6.36 ± 2.305	4.66 ± 0.530	6.79 ± 0.552
PG 32:1	0.33 ± 0.028	0.22 ± 0.057	0.33 ± 0.085	0.25 ± 0.049	0.28 ± 0.198	0.33 ± 0.148
PG 33:1	0.40 ± 0.085	0.15 ± 0.014	0.29 ± 0.177	0.31 ± 0.106	0.31 ± 0.028	0.32 ± 0.134
PG 32:2	0.06 ± 0.042	0.03 ± 0.014	0.08 ± 0.064	0.04 ± 0.049	0.04 ± 0.021	0.05 ± 0.007
PG 34:1	2.30 ± 0.417	1.62 ± 0.580	2.55 ± 0.834	2.71 ± 0.502	1.49 ± 0.156	2.09 ± 0.078
LysylPG 31:0	18.99 ± 2.058	14.31 ± 4.045 *	22.96 ± 1.846	20.76 ± 2.927	26.86 ± 8.577	18.44 ± 1.669
LysylPG 32:0	27.15 ± 6.314	22.96 ± 8.061	15.09 ± 1.032	22.01 ± 3.366	9.73 ± 1.768	28.00 ± 10.628
LysylPG 32:1	21.32 ± 3.776	46.13 ± 1.930	27.47 ± 8.902	31.31 ± 5.148	41.82 ± 14.121	21.47 ± 1.103

**Table 2:** The influence of the tested antimicrobials dosed alone and in combination on the phospholipid profile of *P. aeruginosa* ATCC 27853. Antimicrobials were used at the following concentrations: 0.78 µg/mL of AgNPs, 0.0313 µg/mL of ciprofloxacin, and 0.125 μg/mL of gentamicin. Asterisk * (*p* < 0.05) indicates values that differ significantly from the control.

Phospholipid Species	Growth Control	AgNPs	Ciprofloxacin	Gentamicin	AgNPs + C	AgNPs + G
PC 34:0	0.47 ± 0.059	0.50 ± 0.003	0.27 ± 0.0176	0.27 ± 0.004	0.45 ± 0.076	0.27 ± 0.092
PC 34:1	1.55 ± 0.178	1.61 ± 0.010	1.28 ± 0.001	1.39 ± 1.588	1.46 ± 0.267	0.74 ± 0.272
PC 34:1	0.23 ± 0.022	0.10 ± 0.000 *	0.12 ± 0.000 *	0.09 ± 0.001 *	0.16 ± 0.055	0.11 ± 0.024 *
PC 36:2	0.10 ± 0.014	0.06 ± 5.034	0.04 ± 0.004	0.09 ± 0.000	0.07 ± 0.016	0.07 ± 0.010
PE 28:0	0.01 ± 0.010	0.02 ± 4.155	0.01 ± 2.663	0.01 ± 2.510	0.01 ± 0.002	0.02 ± 0.001
PE 30:0	0.37 ± 0.017	0.38 ± 0.002	0.34 ± 6.527	0.37 ± 0.000	0.38 ± 0.019	0.42 ± 0.007
PE 30:1	0.07 ± 0.009	0.08 ± 0.001	0.05 ± 4.326	0.07 ± 7.344	0.07 ± 0.011	0.09 ± 0.004
PE 31:0	0.06 ± 0.001	0.07 ± 1.933	0.07 ± 0.000	0.07 ± 7.763	0.06 ± 0.003	0.06 ± 4.997
PE 31:1	0.02 ± 0.004	0.02 ± 0.000	0.01 ± 2.730	0.02 ± 5.417	0.02 ± 0.001	0.02 ± 0.003
PE 32:0	4.93 ± 0.047	4.70 ± 0.204	5.05 ± 0.104	5.07 ± 0.195	4.66 ± 0.191	5.23 ± 0.022 *
PE 32:1	4.51 ± 0.068	4.28 ± 0.810	4.30 ± 0.215	4.66 ± 0.005	4.73 ± 0.376	5.54 ± 0.305 *
PE 32:2	0.47 ± 0.009	0.50 ± 0.016	0.33 ± 8.678 *	0.41 ± 0.000	0.48 ± 0.061	0.46 ± 0.007
PE 33:1	0.13 ± 0.004	0.13 ± 1.358	0.14 ± 0.000	0.13 ± 2.713	0.13 ± 0.012	0.13 ± 0.011
PE 33:2	0.18 ± 0.002	0.18 ± 0.000	0.181 ± 0.000	0.17 ± 1.125	0.23 ± 0.022	0.16 ± 0.000 *
PE 34:1	41.41 ± 0.681	39.54 ± 0.500	44.36 ± 2.384	40.62 ± 0.886	40.73 ± 1.352	35.64 ± 0.914 *
PE 34:2	16.01 ± 0.934	15.47 ± 0.010	15.58 ± 0.024	15.64 ± 0.000	16.27 ± 0.089	14.97 ± 0.849
PE 35:1	0.16 ± 0.009	0.15 ± 2.215	0.15 ± 0.000	0.16 ± 4.219	0.11 ± 0.046	0.13 ± 0.013
PE 35:2	1.02 ± 0.086	0.98 ± 0.061	1.19 ± 2.239	1.05 ± 0.000	0.97 ± 0.094	0.78 ± 0.026
PE 36:2	1.81 ± 0.101	1.67 ± 0.008	1.74 ± 0.031	1.69 ± 0.004	1.82 ± 0.011	1.53 ± 0.145
PG 31:1	0.01 ± 0.003	0.01 ± 3.656	0.01 ± 1.0389	0.01 ± 1.228	0.01 ± 0.000	0.01 ± 0.004
PG 32:0	2.10 ± 0.047	2.18 ± 0.001	2.07 ± 0.012	2.43 ± 0.037	2.06 ± 0.005	3.19 ± 0.053 *
PG 32:1	2.21 ± 0.050	2.26 ± 0.188	1.86 ± 0.039	2.30 ± 0.015	2.23 ± 0.186	3.32 ± 0.296 *
PG 32:2	0.27 ± 0.007	0.24 ± 0.002	0.19 ± 0.004	0.23 ± 6.767	0.24 ± 0.043 *	0.27 ± 0.037
PG 33:1	0.07 ± 0.004	0.09 ± 9.740	0.07 ± 2.156	0.07 ± 1.903	0.07 ± 0.007	0.09 ± 0.002 *
PG 34:1	14.09 ± 0.792	16.32 ± 6.014	14.10 ± 0.392	15.66 ± 1.571	14.69 ± 0.834	17.50 ± 1.027
PG 34:2	6.95 ± 0.495	7.61 ± 0.051	5.75 ± 0.003	6.53 ± 0.036	7.06 ± 0.078	8.37 ± 1.129
PG 36:2	0.75 ± 0.012	0.86 ± 3.481	0.75 ± 0.000	0.81 ± 4.572	0.83 ± 0.054	0.87 ± 0.093

**Table 3:** The influence of tested antimicrobials dosed alone and in combination on fatty acid profile of *S. aureus* ATCC 29213. Antimicrobials were used in following concentrations: 3.125 µg/mL of AgNPs, 0.0625 µg/mL of ciprofloxacin, and 0.0078 µg/mL of gentamicin. Asterisk * (*p* < 0.05) indicates values that differ significantly from the control.

Fatty Acid Species	Growth Control	AgNPs	Ciprofloxacin	Gentamicin	AgNPs + C	AgNPs + G
i-14:0	1.13 ± 0.003	1.22 ± 0.007	0.60 ± 0.138	1.41 ± 0.820	1.31 ± 0.108	1.23 ± 0.091
14:0	0.94 ± 0.016	1.59 ± 0.374	1.29 ± 0.400	1.99 ± 0.398 *	1.53 ± 0.086 *	1.33 ± 0.338
i-15:0	8.760.301	8.16 ± 0.206	7.66 ± 0.921	7.68 ± 0.198	7.89 ± 0.003	8.80 ± 1.218
a-15:0	40.98 ± 2.523	35.51 ± 1.320	37.66 ± 1.482	34.27 ± 4.277 *	36.81 ± 0.543	37.34 ± 3.455
15:0	0.82 ± 0.003	0.78 ± 0.057	0.85 ± 0.020	0.92 ± 0.006	0.86 ± 0.019	0.83 ± 0.031
16:0	1.08 ± 0.007	1.21 ± 0.081	1.08 ± 0.077	1.12 ± 0.014	1.15 ± 0.093	1.15 ± 0.021
i-17:0	3.44 ± 0.064	3.42 ± 0.357	2.98 ± 0.186	2.92 ± 0.029	3.14 ± 0.180	3.67 ± 0.494
a-17:0	9.80 ± 0.434	8.83 ± 0.433	8.99 ± 0.113	8.29 ± 0.554	8.67 ± 0.679	9.58 ± 0.880
17:0	2.08 ± 0.001	1.97 ± 0.261	2.06 ± 0.204	2.08 ± 0.021	2.00 ± 0.010	1.95 ± 0.065
18:0	15.68 ± 7.019	25.78 ± 0.903 *	22.66 ± 0.100	28.44 ± 5.714 *	24.15 ± 1.237	21.10 ± 6.358
i-19:0	2.18 ± 0.004	1.82 ± 0.361	1.88 ± 0.275	1.58 ± 0.186 *	1.87 ± 0.175	2.08 ± 0.189
a-19:0	4.95 ± 1.998	3.85 ± 0.609	4.53 ± 0.848	3.63 ± 0.482 *	4.01 ± 0.359	4.34 ± 0.300
19:0	4.13 ± 0.032	3.07 ± 0.478	3.90 ± 0.793	2.89 ± 0.696 *	3.45 ± 0.278	3.28 ± 0.095
20:0	4.05 ± 0.044	2.78 ± 0.289 *	3.87 ± 1.089	2.76 ± 0.523 *	3.16 ± 0.391	3.34 ± 0.168

**Table 4:** The influence of the tested antimicrobials dosed alone and in combination on the fatty acid profile of *P. aeruginosa* ATCC 27853. Antimicrobials were used at the following concentrations: 0.78 µg/mL of AgNPs, 0.0313 µg/mL of ciprofloxacin, and 0.125 µg/mL of gentamicin. Asterisk * (*p* < 0.05) indicates values that differ significantly from the control.

Fatty Acid Species	Growth Control	AgNPs	Ciprofloxacin	Gentamicin	AgNPs + C	AgNPs + G
8:0	0.24 ± 0.093	0.17 ± 0.007	0.35 ± 0.058	0.20 ± 0.036	0.219 ± 4.276	0.24 ± 0.048
i-12:0	0,00 ± 0.000	0.06 ± 0.088	0.14 ± 0.191	0.16 ± 0.027 *	0.17 ± 0.003 *	0.00 ± 0.000
14:0	1.43 ± 0.217	1.23 ± 0.191	1.27 ± 0.160	1.12 ± 0.044	1.24 ± 0.122	1.34 ± 0.033
i-15:0	0.46 ± 0.014	0.46 ± 0.033	0.41 ± 0.015	0.41 ± 0.025	0.43 ± 0.033	0.45 ± 0.012
16:0	40.84 ± 0.0460	40.26 ± 1.345	42.40 ± 1.046	41.65 ± 0.586	40.27 ± 0.496	40.16 ± 0.570
16:1	8.83 ± 0.273	9.62 ± 1.071	7.78 ± 0.861	8.87 ± 0.604	9.25 ± 0.410	10.32 ± 0.508
17:0	0.61 ± 0.143	0.52 ± 0.006	0.61 ± 0.081	0.48 ± 0.014	0.55 ± 0.021	0.57 ± 0.029
18:0	11.39 ± 2.360	7.10 ± 0.414	11.48 ± 2.075	7.82 ± 1.3120	9.02 ± 0.374	8.87 ± 0.573
18:1	36.19 ± 2.600	39.69 ± 0.274	35.57 ± 2.765	39.29 ± 1.390	38.86 ± 0.466	38.06 ± 0.757

## Data Availability

Dataset available on request from the authors.
